# Making Cavities History: A Global Policy Consensus for Achieving a Dental Cavity–Free Future

**DOI:** 10.1177/23800844211020298

**Published:** 2021-05-24

**Authors:** N.B. Pitts, C. Mayne

**Affiliations:** 1 Alliance for a Cavity-Free Future, King’s College London, London, UK

**Keywords:** caries, public health, dental public health, prevention, caries treatment, evidence-based dentistry/health care

## Abstract

These global consensus recommendations on caries and cavities, as well as evidence that caries is the most prevalent noncommunicable disease (NCD) globally, should be shared throughout institutional, health care professional, industrial, civil society, and patient communities so that the recommendations can be incorporated into policies for achieving a dental cavity-free future. Appropriate inclusion within strategies and action plans globally and locally will accelerate progress toward Making Cavities History, as well as improving NCDs and wider health.

Dental caries is the world’s most prevalent noncommunicable disease (Marcenes et al. 2013). Caries and cavities affect sufferers across the life course (Selwitz et al. 2007; Pitts et al. 2017) and globally are responsible for the largest burden of all disease (Marcenes et al. 2013).

Untreated caries and cavities can adversely affect the quality of life of sufferers in multiple ways (Pitts et al. 2017). Untreated caries also creates sizable economic challenges with huge global costs. Caries is a widespread problem and has the largest impact of any oral health issue, yet the burden and pain caused by caries and cavities are preventable (Selwitz et al. 2007).

By tackling caries at the early stages, we can avoid cavities. This can not only lead to improvements in oral health but can also, through common risk factors, go a long way toward tackling other major (and costly) noncommunicable diseases, for wider health benefits (Fig. 1).

**Figure 1.**
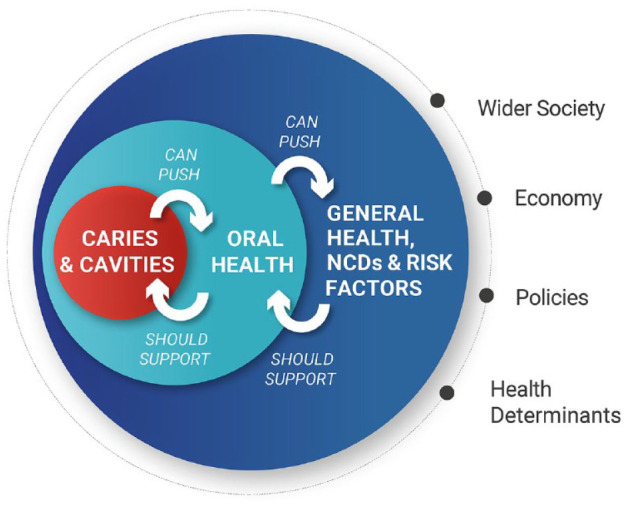
Interrelationships between caries and cavities, oral health, and wider health.

Despite this evidence, caries is typically ignored in health policy. Previous global policy recommendations have not explicitly mentioned caries, and caries is not currently visible in global or national noncommunicable disease (NCD) strategies. This means that it is invisible to health policymakers and does not attract available resources.

We can demonstrate that a cavity-free future is possible and also widely desirable. With this shared international vision for the past decade, we are now at a turning point, with dental authorities around the world united, speaking with one voice to push for change (Pitts et al. 2017; Varenne and Fox 2021) and the avoidance of the restoration replacement spiral (Elderton 1990) (Fig. 2).

**Figure 2.**
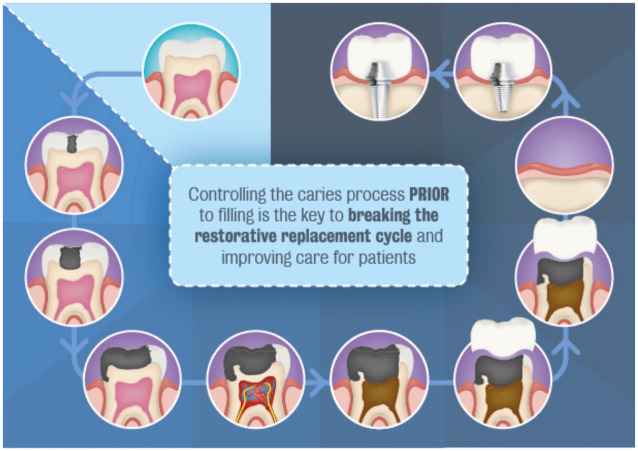
The restorative replacement spiral.

Making progress with caries requires both World Health Organization (WHO)–level global policy agreements (WHO 2021) and country-level policy implementation. The Alliance for a Cavity Free Future (ACFF) Taskforce was formed in 2020 to bring together world-leading experts in cariology, behavior change, public health, and health policy. An intensive series of virtual meetings was held between October and December 2020 to achieve a consensus as to the required direction of travel and to create policy recommendations, looking to ensure international-level agreement and buy-in as well as locally suitable frameworks for initiating effective policy and systems development.

The key outcomes of the taskforce were that population education and behavior must drive change around primary NCD prevention, with caries recognized in global and national NCD agendas. It is critical to tackle sugar and the other major dietary risk factors for NCDs while continuing to educate people on the importance of nutrition and hygiene. To move toward a cavity-free future, there needs to be integrated and incentivized primary and secondary caries prevention across the life course as part of wider health provision. We also need to push to ensure the availability of systematic surveillance data to monitor actions and progress.

The following recommendations are a consensus co-created by international representatives from across the global dental community. The authors strongly believe that if a concerted, global effort is made, dental caries can be stopped in its tracks. We call upon policymakers to consider these recommendations so that we might feasibly create a future free from cavities.

## Policy Recommendations to Make Cavities History

The task ahead will be to align systems, develop new tools and reimbursement incentives, and form a collaborative approach to primary oral care while ensuring recognition of the severity of the issue at hand from those with the power to promote change. Making progress requires both global-level policy agreements and country-level policy implementation. The following are the consensus policy recommendations agreed by the taskforce. These recommendations are offered by the taskforce to policymakers as a springboard for ensuring that caries and cavities are given a platform within health policy discussion, with a view to successfully influencing improvements in oral and general health (Fig. 1).

### 1. Population and Health Professional Education and Behavior Must Drive Change around Primary Prevention of NCDs

The recognition of caries as a manageable NCD by policymakers, professionals, and the public, as well as the implementation of suitable oral health education programs for all stakeholder groups, will be essential in driving a shift in attitude toward and improvements in dental caries.

#### I. Effective prevention and management of dental caries and cavities across the life course

Oral diseases, notably dental caries and cavities, are largely preventable. Disease prevention and management allow individuals to live a pain-free and high-quality life throughout their lives and reduce the impact on health care expenditure.

We call upon policymakers:

- To recognize caries and cavities in their national NCD plans at the same level as other major NCDs, such as diabetes, which share common risk factors
- To implement oral health education programs in preschools and schools for both students and parents, with the support of key stakeholders from across the health care spectrum, based on best practice examples from across the world, such as the Childsmile program
- To implement oral health education programs specifically addressed at vulnerable groups, such as pregnant women, the elderly population, and so on
- To include oral health curricula prevention and management modules as part of the formal and lifelong training of health care professionals across the spectrum

### 2. It Is Critical to Tackle Sugar and Other Major Risk Factors for NCDs

The availability of and education surrounding appropriate nutrition and hygiene is a key element in the fight against dental caries. The dangers of frequent sugar consumption, particularly for children under 2 y old, must be addressed through multiple care routes to ensure coverage is broad. By suitably addressing these risk factors, benefits will be achieved across multiple areas of health with an increased awareness of factors fundamental to achieving health across the life course.

#### II. Addressing caries and cavities risk factors across the life course to fight major NCDs

Sugar consumption is one of the most common dietary risk factors across the life course toward developing an NCD, notably diabetes, cardiovascular diseases, cancer, and obesity, and results in massive public expenditures for the treatment of caries and cavities. Reduction in the intake of sugar-sweetened beverages and foods is advised globally as part of healthier dietary patterns to help reduce energy intake, the risk of obesity, and obesity-related disorders.

We call upon countries:

- To include oral diseases, particularly caries and cavities, in their national NCD plans when targeting high sugar consumption in major NCDs. In turn, directly addressing caries and cavities will also address the major NCDs.

We encourage policymakers to focus on:

- Creating effective solutions, with all stakeholders, to provide affordable and accessible healthy food and drinkable water as well as decrease the purchase of sugared food or drinks through taxation policies
- The importance of decreasing sugar intake in the first 2 y of life as these years are crucial in determining a child’s well-being in adulthood
- The implementation of oral hygiene education programs in primary and secondary school, including nutrition programs modeling healthy and affordable nutrition and hygiene practices
- The promotion of healthy food consumption across the life course, notably in schools and in the workplace

### 3. There Needs to Be Integrated Primary and Secondary Caries Prevention across the Life Course

The integration of caries and cavities into wider oral health policies and continuing the push toward including prevention within national health programs as an essential part of universal health coverage is key to making cavities history. We call for greater access to integrated primary and secondary preventive dental care to maintain health. As part of policy development, consideration must be given to ensuring effective systems are in place to support preventive dentistry and the development of the local workforce delivering care. Achieving effective caries prevention and management across the life course will involve educating and empowering the existing dental workforce to deliver up-to-date care pathways, as well as restructuring health systems to allow for effective continued development in best practice for caregivers. It also means expanding, where possible, the range of people who can advise and refer patients and, in some cases, treat basic dental health needs to increase care accessibility.

#### III. Integration of primary and secondary prevention across the life course to address the burden of cavities and caries

There is no general health without oral health. Therefore, a focus on prevention across the life course is key.

We call upon policymakers:

- To ensure a shift toward optimal standards of care and preventive dental medicine, which is outcomes oriented and based on best practice implementation and includes access to affordable future innovations in caries prevention technologies and care delivery, in discussion with all key stakeholders from across the health care spectrum. This should furthermore address the reduction of the environmental footprint through the reduction of the use of dental amalgam, in line with the implementation of the Minamata Convention on Mercury, and other restorative materials.
- To ensure the implementation, access, and affordability of proven preventive measures, such as public health use of fluoride and effective and affordable fluoride toothpaste, to promote and preserve oral health, in discussion with all key stakeholders from across the health care spectrum
- To strengthen the interconnectivity between oral and general health through a holistic approach that integrates oral health into general health promotion strategies as well as in academic curricula and lifelong learning for professionals
- To integrate equitable and affordable access to essential care for the most common dental needs in primary care services (under universal health coverage) to improve the prevention and management of NCDs as well as caries and cavities
- To integrate oral health policies, particularly the inclusion of caries and cavities prevention policies, into national health programs as cost-effective measures and part of primary care services, as well as to translate them into national prevention programs. The focus should be on the entire life course and range from early childhood caries to healthy aging, in line with the WHO Decade of Healthy Ageing.

### 4. We Need Systemic Surveillance Data to Monitor Actions and Progress

So that we can effectively map caries incidence and monitor the success of different approaches to eliminating cavities, multinational surveillance programs for data on the prevalence and incidence of caries and cavities are essential. Building an aligned system for reporting from countries of high, low, and emerging economic status will help to offer a more comprehensive overview of the true burden of caries globally and in turn will help us to monitor successful interventions over the medium and long term in our battle against cavities. This will then allow for effective evaluation and adjustment of programs and policies, informing future decisions based on examples of best practice emerging from the programs. A clear, universal reporting language must be used in the creation of these programs to ensure that the data exchanged are useful and comprehensible and to minimize the margin for error within reporting.

#### IV. Comprehensive data collection for effective prevention and management of dental caries and cavities

The lack of data, in general, as well as the lack of consistent data on dental caries and cavities, does not allow for proper decision making to ensure effective strategies in the prevention and management of dental caries and cavities.

We call upon policymakers:

- To create a sustainable public surveillance program for the collection of data on the prevalence and incidence of caries and cavities across the life course, taking best practice examples as a starting point
- To monitor progress and evaluate the impact of policies aimed at preventing and managing caries and cavities
- To use the same case definition and exchange data on current oral health policies
- To establish a monitoring system to ensure implementation of the above recommendations

Steps should also be taken both at an international level and within countries to start to collect appropriate data (with prevalence/disease data that include initial-stage disease) that will be needed for the long-term assessment of the costs and impacts of caries management and cavity prevention. The potential of expanding existing NCD frameworks to include surveillance and monitoring of caries risk factors, as well as caries prevalence and burden, should be explored as an efficient route to achieving results across the NCD agenda. The collection of these data will be a big step in accelerating and refining progress toward a cavity-free future.

## Author Contributions

N.B. Pitts, contributed to conception, design, data acquisition, analysis, and interpretation, drafted and critically revised the manuscript; C. Mayne, contributed to data interpretation, drafted and critically revised the manuscript. Both authors gave final approval and agree to be accountable for all aspects of the work.
